# Is the promotion of research reflected in bibliometric data? A network analysis of highly cited papers on the Clusters of Excellence supported under the Excellence Initiative in Germany

**DOI:** 10.1007/s11192-016-1925-2

**Published:** 2016-03-23

**Authors:** Lutz Bornmann

**Affiliations:** Division for Science and Innovation Studies, Administrative Headquarters of the Max Planck Society, Hofgartenstr. 8, 80539 Munich, Germany

**Keywords:** Excellence Initiative, Cluster of Excellence, Bibliometrics, Highly cited papers

## Abstract

Under the Excellence Initiative, a number of Clusters of Excellence in Germany have been supported since 2006 and 2007—including each a limited number of cooperating institutions. The aim of the present study is to investigate whether support for Clusters of Excellence since 2006 and 2007 is reflected in bibliometric network data. For this purpose, a comparison is made between network data in the period before support started (2003–2005) and in the period after support started (2009–2011). For these two periods, a co-authorship network is generated (based on the funded institutions). This is based on publications which are among the 1 % most frequently cited publications in their respective fields and publication year and which have at least one author from Germany. As the results show, the outcomes this yields for life sciences and natural sciences differ from each other. Whereas natural sciences display an effect of establishment of Clusters of Excellence on the bibliometric networks, this was not true of life sciences. After establishment of the Clusters of Excellence, the network in natural sciences not only contained more institutions of a Cluster of Excellence, but these institutions were distributed across fewer bibliometric clusters in the network than before establishment. In other words the structure of the Clusters of Excellence was better reflected in the network.

## Introduction

The promotion of excellence in research is a vital goal of many national science policies. For this purpose, a number of countries (including Australia, Belgium, France, Italy, New Zealand, and UK) have already established national evaluation systems (Bornmann 2015), which subject research institutions to continuous evaluation. Whereas these systems focus mainly on an ex-post evaluation of research at institutions, in other countries, like South Korea and Germany, ex-ante evaluations are conducted for the award of research grants in order to support a small number of institutions (Hur and Bessey 2013; Schweizerischer Wissenschafts- und Technologierat 2013). For example, the Excellence Initiative was launched in Germany in 2006, which provided a total of €1.9 billion in additional funding for three funding lines between 2006 and 2011: (1) Graduate schools to promote early career researchers; (2) Clusters of Excellence to promote top-level research; and (3) institutional strategies to promote top-level university research. The Excellence Initiative was intended to break up the often-cited homogeneity among the institutions of the German university system (Hur and Bessey 2013).

In recent years, many studies have been published which investigated the validity of funding decisions (like those which led to the promoted institutions of the Excellence Initiative). Since these studies are generally based on bibliometric data, Wouters et al. (2015) present a comprehensive overview of these studies under the title “correlating bibliometrics with peer review”. These studies investigated peer review processes (and the resulting decisions) in three main areas: peer review of journal manuscripts, peer review of applications for funding and career promotions, and national peer-review based assessments. Wouters et al. (2015) summarize the results of the studies as follows: “The results of peer review-based decisions generally show positive correlations to selected bibliometric performance data. However, it matters a lot exactly which forms of peer review and which specific dimensions of peer review are being related to exactly which bibliometric indicators” (p. 65). In other words, the studies show a tendency in their results, but there are also greater variations in the results. Bornmann (2011) who published an overview of studies investigating (bibliometrically) journal and grants peer review processes points to the fact that the success of funding decisions should be investigated properly (by using bibliometric data). It is only possible with the results of these studies to decide whether the goals of funding lines have been reached, which funding lines should be continued and how future funding lines should be designed.

This study undertakes a bibliometric analysis of one funding line of the Excellence Initiative: Clusters of Excellence to promote top-level research. It is the general aim of all three funding lines (1: Graduate schools to promote early career researchers; 2: Clusters of Excellence to promote top-level research; 3: Institutional strategies to promote top-level university research) to achieve top positions in international rankings for a limited number of German institutions (especially of the third line). However, it is not the intention of this study to investigate whether the general goal was really reached. This study is intended to investigate whether the structure given by the second funding line is reflected in bibliometric data. The DFG describes the second funding line as follows: “Clusters of Excellence will enable German university locations to establish internationally visible, competitive research and training facilities, thereby enhancing scientific networking and cooperation among participating institutions. Clusters of Excellence should form an important part of a university’s strategic and thematic planning, significantly raise its profile and reflect its considered long-term priorities. They should also create excellent training and career conditions for young researchers” (http://www.dfg.de/en/research_funding/programmes/excellence_initiative/clusters_excellence/index.html). Accordingly, the aim of providing support for Clusters of Excellence is to create internationally competitive centres of research (including selected cooperating institutions) (Hur and Bessey 2013). The institutions supported under this funding line are referred to as excellence institutions in the following sections.

### Evaluation of the Excellence Initiative

What form has the evaluation of the Excellence Initiative in Germany taken so far and what evaluations are planned? The results of a wide-ranging evaluation of the Excellence Initiative and its effects on the German science system were published in 2016. The results of the evaluation are as follows: "The Excellence Initiative has made the German university system more dynamic and has become a tangible symbol for the will to improve the international competitiveness of German universities. To achieve that goal, the Excellence Initiative has given additional financial means to the best performing universities in order to strengthen their research and to optimise their organisational structures. Thus, the opinion of the IEKE [Internationale Expertenkommission Exzellenzinitiativ] about the usefulness of the Excellence Initiative is very positive" (Internationale Expertenkommission zur Evaluation der Exzellenzinitiative 2016, p. 5). The Excellence Initiative was monitored regularly by the Institute for Research Information and Quality Assurance (iFQ) until 2012. The results and publications obtained from this project can be viewed on the following web site: www.research-information.de/Projekte/projekte_container.php?id=ExzellenzXXXprojekte_exzellenz.html. By way of example, under this project all experts involved in the appraisal of projects on two funding lines of the Excellence Initiative (graduate schools and Clusters of Excellence) were questioned in a wide-ranging study. Its task was to appraise and evaluate the suitability and appropriateness of the peer review procedure (Möller et al. 2012).

In one bibliometric study, Mittermaier (2011) showed that in most of the nine funded excellence universities (in the funding line: Institutional strategies to promote top-level university research) the increase in publications displayed an above-average rate of increase for German universities. In addition to the study by Mittermaier (2011), it was possible to research only two further studies on the Excellence Initiative in the Web of Science (WoS, Thomson Reuters) literature database: Kegen (2015) evaluated network data of female and male investigators of two research institutions in the Excellence Initiative. Using case studies in the field of graduate schools funded by the Excellence Initiative, Bloch et al. (2014) investigated stratificatory efforts that are connected to education at universities.

The aim of the present study is to investigate whether support for Clusters of Excellence since 2006 and 2007 is reflected in bibliometric network data. For this purpose, a comparison is made between network data in the period before support started (2003–2005) and in the period after support started (2009–2011). For these two periods, a co-authorship network is generated (based on the institutions of the respective authors and not the authors’ names). This is based on publications which are among the 1 % most frequently cited publications in their respective fields and publication year and which have at least one author from Germany. A comparison of the networks in the periods before and after the support started should show whether the structure of Clusters of Excellence is reflected in the bibliometric data. The anticipated result would be that, after the support started, there would not only be more excellence institutions in the network than before support, but also that the institutions of a Cluster of Excellence would cluster more prominently in the network.

## Methods

### Data on the Clusters of Excellence in the Excellence Initiative

The information on the institutions in the Clusters of Excellence was researched in the DFG database (in May 2015): http://www.dfg.de/en/research_funding/programmes/list/index.jsp?id=EXC. In this study, the analysis only covered Clusters of Excellence (or institutions) which had been funded since 2006 or 2007 in natural and life sciences (as they had been assigned by the DFG to the two disciplines). The issue here is therefore the first two rounds of the Excellence Initiative. The Clusters of Excellence funded since 2012 (third round of the Excellence Initiative) are not included in this study because at the time of analysis in 2015 the citation window for the impact analysis was not long enough (Wang 2013). Also, no Clusters of Excellence from the fields of “humanities and social sciences” and “engineering sciences” were included, as one cannot expect that a bibliometric analysis in these fields leads to reliable and valid results (Moed 2005). In the DFG database, all institutions were searched for the Clusters of Excellence in life sciences and natural sciences. The search referred to the institution of the coordinator of the Cluster of Excellence, the applicant institution and the participating institutions.

 Tables 1 and 2 show the excellence institutions mentioned in the database on the Clusters of Excellence for the years 2006 and 2007. Each Cluster is denoted in the tables by an abbreviation, which is then used in the following sections of the paper instead of the full name of the Cluster.

**Table 1:** Institutions funded in a Cluster of Excellence in the field of physical sciences since 2006 and 2007

Cluster^a^	Institution	Institution	Institution	Institution	Institution
NIM	Bayerische Akademie der Wissenschaften	Deutsches Museum	Helmholtz-Zentrum für Umweltforschung	MPI für Biochemie	MPI für Quantenoptik
MATHE	U Bonn	MPI für Mathematik			
OCEAN	U Kiel	GEOMAR Helmholtz-Zentrum für Ozeanforschung Kiel	Institut für Weltwirtschaft an der Universität Kiel	Muthesius-Kunsthochschule	
UNIV	TU München	U München	Bayerische Akademie der Wissenschaften	Europäische Südsternwarte	MPI für Astrophysik
MAP	MPI für Quantenoptik	TU München	U München	Helmholtz Zentrum München	U der Bundeswehr München
CLISAP	U Hamburg	Deutsches Klimarechenzentrum	Helmholtz-Zentrum Geesthacht	MPI für Meteorologie	
MARUM	U Bremen	Alfred-Wegener-Institut Helmholtz-Zentrum für Polar- und Meeresforschung	Leibniz-Zentrum für Marine Tropenökologie	MPI für Marine Mikrobiologie	Senckenberg Forschungsinstitut und Naturmuseum
CATAL	TU Berlin	Fritz-Haber-Institut der Max-Planck-Gesellschaft	MPI für Kolloid- und Grenzflächenforschung	FU Berlin	HU Berlin

Cluster^a^	Institution	Institution	Institution	Institution
NIM	U München	U Augsburg		
MATHE				
OCEAN				
UNIV	MPI für Physik	MPI für Plasmaphysik	MPI für extraterrestrische Physik	Halbleiterlabor
MAP				
CLISAP				
MARUM	Jacobs University Bremen			
CATAL	U Potsdam			

^a^Clusters of Excellence: Nanosystems Initiative Munich (NIM), Mathematics: Foundations, Models, Applications (MATHE), Future Ocean (OCEAN), Origin and Structure of Universe (UNIV), Munich-Centre for Advanced Photonics (MAP), Integrated Climate System Analysis and Prediction (ClISAP), Ocean in Earth System/Center for Marine Environmental Sciences (MARUM), Unifying Concepts in Catalysis (CATAL)

**Table 2:** Institutions funded in a Cluster of Excellence in the field of life sciences since 2006 and 2007

Cluster^a^	Institution	Institution	Institution	Institution
REBIRTH	Medizinische Hochschule Hannover	U Hannover	Fraunhofer-Institut für Toxikologie und Experimentelle Medizin	Friedrich-Loeffler-Institut
NETWORK	U Heidelberg	Deutsches Krebsforschungs-zentrum	European Molecular Biology Laboratory	Heidelberger Institut für Theoretische Studien
CIPSM	U München	TU München	Helmholtz Zentrum München	MPI für Biochemie
MACRO	U Frankfurt	MPI für Biophysik	MPI für Hirnforschung	
CARDIO	U Frankfurt	U Gießen	MPI für Herz- und Lungenforschung	
CRTD	TU Dresden	Max Bergmann Zentrum für Biomaterialien	MPI für molekulare Zellbiologie und Genetik	
NANO	U Göttingen	MPI für biophysikalische Chemie	Leibniz-Institut für Primatenforschung	Klinikum Kassel
CECAD	MPI für Stoffwechsel-forschung	U Köln	Deutsches Zentrum für Neurodegenerative Erkrankungen	MPI für Biologie des Alterns
NEURO	FU Berlin	HU Berlin	Deutsches Rheuma-Forschungszentrum Berlin	Leibniz-Institut für Molekulare Pharmakologie
BIOSS	U Freiburg	Fraunhofer-Institut für Physikalische Messtechnik	MPI für Immunbiologie und Epigenetik	
INTER	U Kiel	U Lübeck	Forschungszentrum Borstel Leibniz-Zentrum für Medizin und Biowissenschaften	MPI für Evolutionsbiologie
CIN	U Tübingen	Deutsches Zentrum für Neurodegenerative Erkrankungen	Fraunhofer-Institut für Produktionstechnik und Automatisierung	Helmholtz Zentrum München

Cluster^a^	Institution	Institution	Institution	Institution
REBIRTH	Helmholtz-Zentrum für Infektionsforschung	Laser Zentrum Hannover	MPI für molekulare Biomedizin	Stiftung Tierärztliche Hochschule Hannover
NETWORK	MPI für medizinische Forschung	Zentralinstitut für Seelische Gesundheit		
CIPSM				
MACRO				
CARDIO				
CRTD				
NANO	Laser-Laboratorium Göttingen	MPI für experimentelle Medizin	XLAB - Göttinger Experimentallabor für junge Leute	
CECAD				
NEURO	Max-Delbrück-Centrum für Molekulare Medizin	Charite		
BIOSS				
INTER	Muthesius-Kunsthochschule			
CIN	MPI für Intelligente Systeme	MPI für biologische Kybernetik		

^a^Clusters of Excellence: From Regenerative Biology to Reconstructive Therapy (REBIRTH), Cellular Networks: From Molecular Mechanisms to Quantitative Understanding of Complex Functions (NETWORK), Center for Integrated Protein Science Munich (CIPSM), Macromolecular Complexes in Action (MACRO), Cardiopulmonary System (CARDIO), Center for Regenerative Therapies Dresden (CRTD), Nanoscale Microscopy and Molecular Physiology of Brain (NANO), Cellular Stress Responses in Aging-associated Diseases (CECAD), NeuroCure—towards a better outcome of neurological disorders (NEURO), Centre for Biological Signaling Studies—from Analysis to Synthesis (BIOSS), Inflammation at Interfaces (INTER), Werner Reichardt Centre for Integrative Neuroscience (CIN)

### Dataset used

The dataset for this study is composed of papers among the 1 % most frequently cited papers in their particular subject category and year of publication. The papers were researched in an in-house database of the Max Planck Society, itself based on the WoS. As there are percentiles, as defined by Thomson Reuters for use in InCites, for all papers in the in-house database, the percentiles form the basis for selection of the 1 % most frequently cited papers: papers with a percentile of ≤1 were selected for data analysis. These papers are referred to below as highly cited papers. As the analysis in this study relates to institutions in Germany, only those papers with at least one author from an institution in Germany, and of these papers only the institutions in Germany, are included in the data analysis.

The DFG has assigned the Clusters of Excellence to life sciences and natural sciences. Also, in order to be able to assign the highly cited papers to these two subject areas, recourse was made to the concordance list of subject categories in the WoS and broad subject areas at https://images.webofknowledge.com/WOKRS56B4/help/WOS/hp_subject_area_terms_easca.html. This shows how the WoS subject categories can be assigned to “life sciences & biomedicine” (here: life sciences) and to “physical sciences” (here: natural sciences). Almost all WoS subject categories of the highly cited papers were allocated to the two broad areas using this concordance list. However, in some cases a revision had to be made: for example, there are subject categories for the highly cited papers which do not exist on the website. These have been re-assigned to life sciences or natural sciences: “Biochemical Research Methods”; “Biology”; “Biology, Miscellaneous”; “Cell & Tissue Engineering”; and “Medicine, Miscellaneous” have been assigned to life sciences and “Geography”; “Geosciences, Multidisciplinary” to natural sciences.

For natural sciences, 1311 affiliations from 957 papers published between 2003 and 2005 and 2090 affiliations from 1238 papers published between 2009 and 2011 were included in the analysis. The analysis for life sciences covers 1823 affiliations in 1781 papers from the years 2003 to 2005 and 3785 affiliations in 2030 papers from the years 2009 to 2011.

### Statistical procedures

The bibliometric data on the two time periods and subject areas was used to create co-authorship networks with the aid of the Pajek software (de Nooy et al. 2011) and VOSviewer (van Eck and Waltman 2010). Here, the networks were not generated at author level but at institution level, in terms of the institutions named by the authors in their publications. Accordingly, the institutions in Germany constitute the nodes in the network and the papers published jointly by two institutions are the links between the nodes. In the networks, a link is established between two institutions if two different institutions in Germany are named in one publication. This also means that only one link is established between the institutions if several authors of the publication are at one of the two institutions. This restriction is intended to prevent a few publications with many authors from one institution having a major impact on the results.

To generate the networks, initially 2-mode datasets were selected from the MPG in-house database where the corresponding institutions were listed for each publication. However, the data on institutions were not taken directly from the WoS, as the WoS does not reliably assign all publications published by an institution to that institution (Haustein and Larivière 2015). Instead, data from the Competence Centre for Bibliometrics (www.bibliometrie.info) is used for the study. This data enables assignment of publications to institutions in Germany with as much completeness, reliability and sustainable usefulness as possible (Winterhager et al. 2014). The 2-mode datasets from the in-house database were entered into Pajek and edited there in preparation for network analysis (for example by conversion of the 2-mode into 1-mode datasets) (Leydesdorff et al. 2014). The network analyses themselves were carried out with VOSviewer. Below, the only networks to be presented will be those generated with VOSviewer. Network analysis in this study is intended to identify those institutions which have formed clusters with particularly frequent cooperation activities.

In order to be able to identify the institutions which belong to a Cluster of Excellence in the visualizations, their names are written in large type and before each excellence institution is the name of the Cluster of Excellence to which the institution belongs. In VOSviewer there are mainly two means of evaluating the cooperation activities engaged in by the institutions: (1) VOSviewer generates two-dimensional distance-based maps, where the distance between two institutions reflects the strength of the relationship between them (van Eck and Waltman 2010, 2014). Accordingly, the closer the positions of the two institutions to each other in the network, the more frequent are their joint publications. (2) The nodes in a network are also assigned by VOSviewer to institutional clusters (they are highlighted in different colours). These clusters identify closely related nodes, where each node is assigned to only one cluster (van Eck and Waltman 2014). VOSviewer uses a modularity-based clustering technique, which is closely related to the multidimensional scaling technique (Waltman et al. 2010) and is based on the smart local moving algorithm (Waltman and Eck 2013). The use of two methods of evaluating the cooperation activities is intended to inspect the reliability of the results: The results should be reliable if both methods lead to similar results.

Apart from analysis of the whole network of institutions which have published highly cited papers in the periods under consideration, an additional analysis with Pajek and VOSviewer is conducted which focuses on the sub-network of the most intensively networked institutions. These intensively networked institutions have been identified with the aid of the k-core technique. “A *k*-*core* is a maximal sub-network in which each vertex has at least degree k within the sub-network” (de Nooy et al. 2011, p. 82). The additional analysis is used to check to what extent the Clusters of Excellence are present in this intensively cross-linked network.

In the networks illustrated below, the 400 strongest normalized links between the institutions are indicated.

## Results

### Results for life sciences

Below, the bibliometric network analysis (based on co-authorship relations) is used to check whether the establishment of Clusters of Excellence in Germany leads to a discernible change in the network structure in those institutions which have published highly cited publications. The results of the network analyses are set out below separately for life sciences and natural sciences. Life sciences are the subject of this section.

**Fig. 1:**
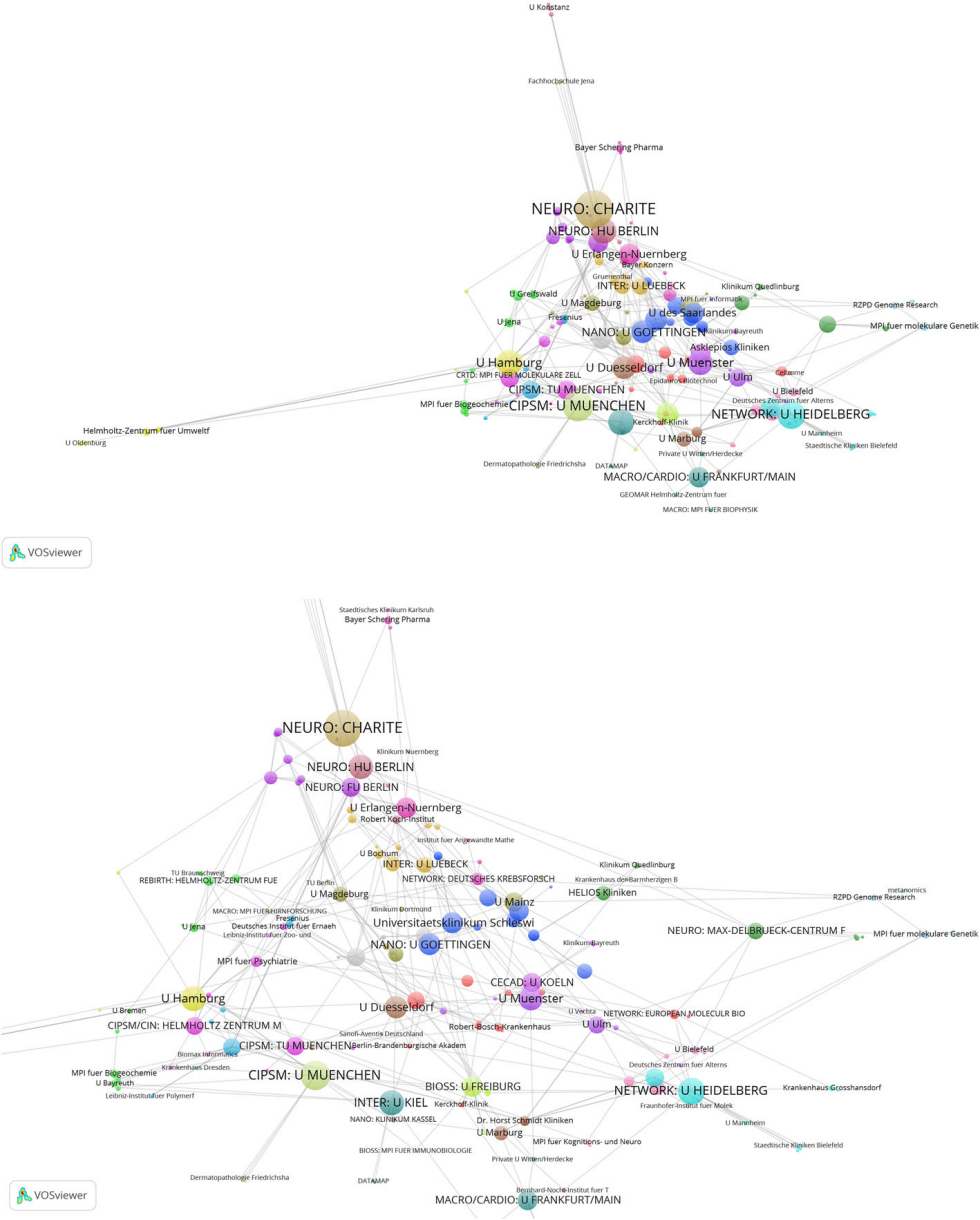
Network of institutions by co-authorships (life sciences, 2003–2005). Whereas the *upper graph* shows all institutions, the *lower graph* focuses on one section of the tightly networked institutions in the *upper graph*. The map and network files are available at doi:10.6084/m9.figshare.1546480. Both files can be opened in VOSviewer

**Fig. 2:**
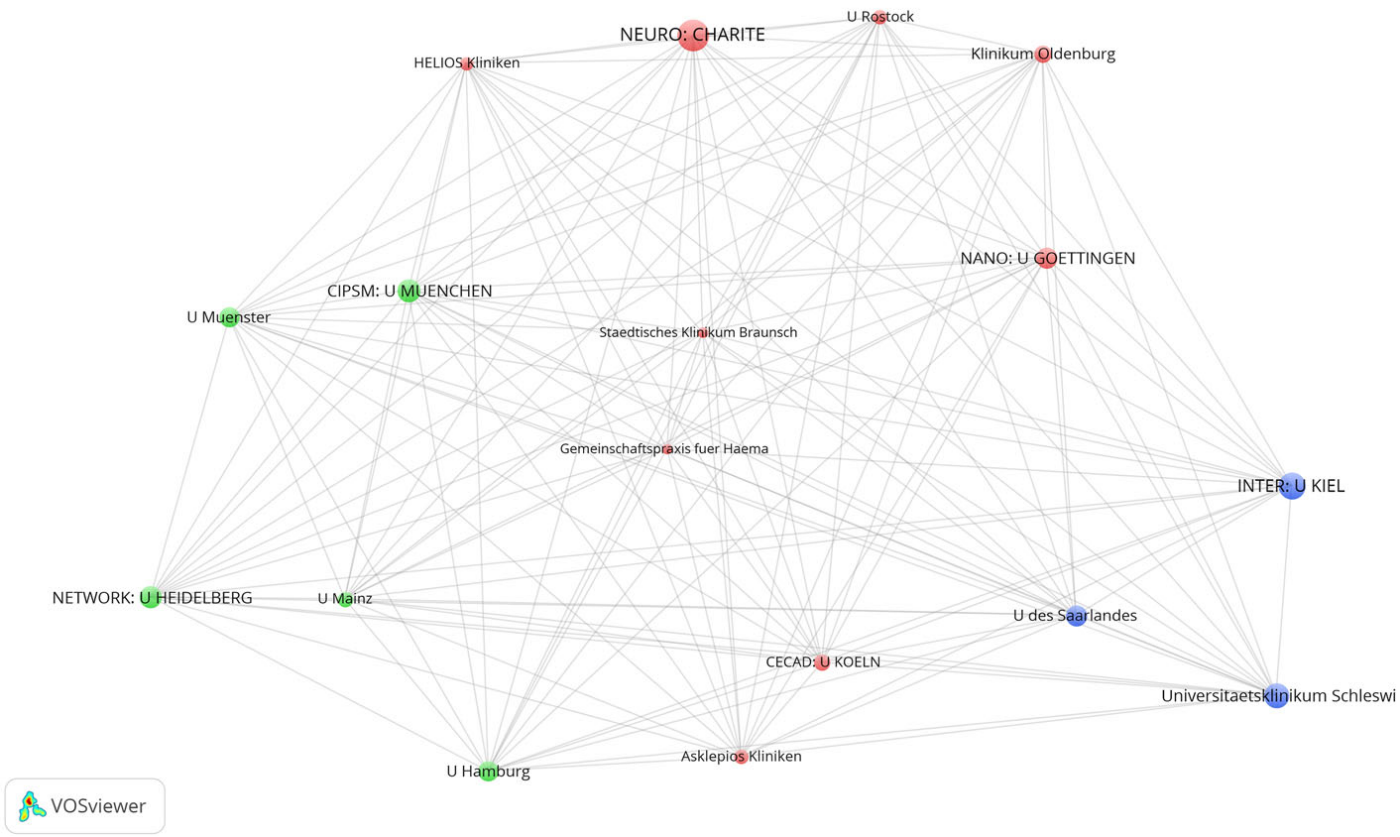
Sub-network of institutions from Fig. 1 which are tightly connected by co-authorships (life sciences, 2003–2005). Each institution has published a minimum of 16 papers with another institution

 Figure 1 shows the network of the institutions which have published at least one highly cited paper in cooperation with another institution in Germany between 2003 and 2005. The institutions are presented in a two-dimensional space such that institutions that have cooperated together frequently are arranged more closely to each other, than those which have rarely cooperated together. As a further means of identifying closely related institutions, the institutions are assigned to clusters which are indicated by different colours. The 186 institutions in total are assigned to 23 clusters. The size of a node reflects the activity of an institution: the bigger the node, the greater the number of papers the institution was involved in. For example, the Charite as the institution with the biggest node has a total link strength of 168; for U München this figure is 108. Figure 2 is a sub-network taken from Fig. 1 with those institutions which have each published at least 16 highly cited papers with another institution. These institutions form the core of the institutions in Fig. 1 which are most tightly networked together.

As Figs. 1 and 2 show, a number of excellence institutions can be found among the institutions which have published at least one highly cited publication between 2003 and 2005. With some institutions of an Excellence Cluster, it can also be seen that they are very tightly positioned in relation to each other in the space, for example TU München and U München in CIPSM. Seen overall however, neither the cluster structure in Figs. 1 and 2 (given by the coloured nodes) nor the positions of the excellence institutions in the visualized networks (which are closer or further away positioned) suggest a structure determined by the Clusters of Excellence.

As a visual inspection of the results in Figs. 1 and 2 is insufficient for an examination of the representative nature and links of the excellence institution in the network, Table 3 quotes the number of excellence institutions which appear or do not appear in the networks (see the numbers in the 2003–2005 section). As the figures in the table show, of the 58 excellence institutions 42 (72 %) are represented in the network of those institutions which have published highly cited papers between 2003 and 2005; 16 institutions are therefore not represented in the network. Also, Table 3 shows the number of network clusters in which the institutions of a Cluster of Excellence are represented and how the ratio of institutions per network cluster looks. For example, six excellence institutions belonging to the REBIRTH Cluster of Excellence are represented in three network clusters. This corresponds to an institutions per network cluster ratio of 0.5. The lower this ratio, the better the network reflects the cooperation determined by the Cluster of Excellence. With a value of 1, each institution of a Cluster of Excellence is in one other network cluster. Across all Clusters of Excellence, the average institutions per network cluster ratio is 0.68.

**Table 3:** The number of excellence institutions which appear or do not appear in the networks (Figs. 1, 2, 3, 4)

Cluster of excellence	Number of institutions	Number of institutions within network	Number of institutions not in network	Number of network clusters	Institutions per network cluster
2003–2005					
REBIRTH	8	6	2	3	0.50
NETWORK	6	5	1	5	1.00
CIPSM	4	4	0	3	0.75
MACRO	3	3	0	2	0.67
CARDIO	3	3	0	2	0.67
CRTD	3	3	0	1	0.33
NANO	7	4	3	4	1.00
CECAD	4	1	3	1	1.00
NEURO	6	5	1	4	0.80
BIOSS	3	2	1	1	0.50
INTER	5	3	2	3	1.00
CIN	6	3	3	3	1.00
Total	58	42	16		0.68^a^
2009–2011					
REBIRTH	8	6	2	5	0.83
NETWORK	6	5	1	4	0.80
CIPSM	4	4	0	4	1.00
MACRO	3	3	0	2	0.67
CARDIO	3	3	0	3	1.00
CRTD	3	2	1	2	1.00
NANO	7	3	4	3	1.00
CECAD	4	3	1	2	0.67
NEURO	6	5	1	3	0.60
BIOSS	3	2	1	1	0.50
INTER^b^	5	3	2	2	0.67
CIN	6	4	2	4	1.00
Total	58	43	15		0.77^a^

Also, the table shows the number of network clusters in which the institutions of a Cluster of Excellence are represented and how the ratio of institutions per network cluster looks

^a^The average of the ratios of institutions per network cluster is calculated as a harmonic mean

^b^Although the MPI for Evolutionary Biology has published highly cited papers, this was not in cooperation with another institution in the network, so it is not included in the analysis

**Fig. 3:**
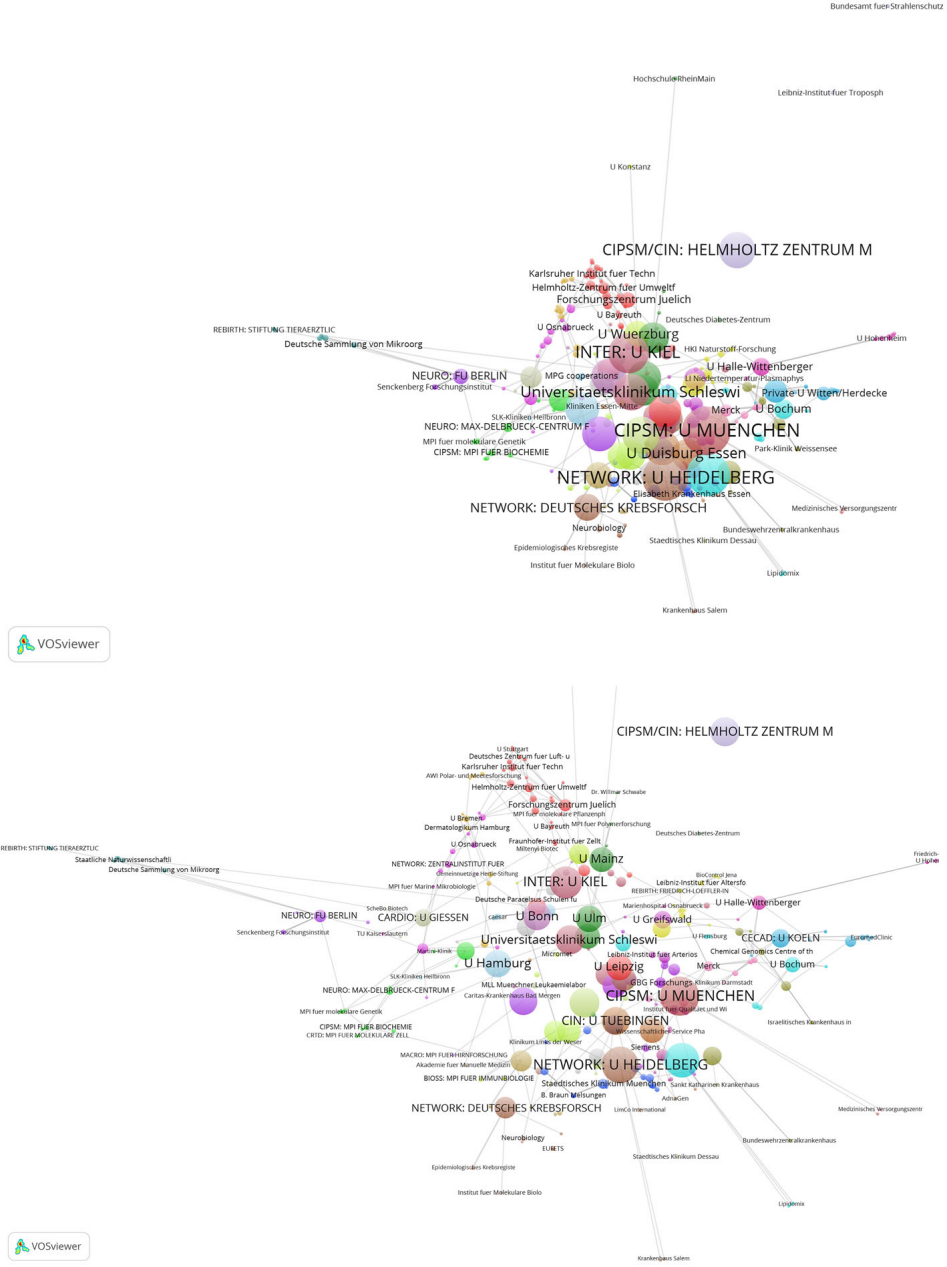
Network of institutions by co-authorships (life sciences, 2009–2011). Whereas the *upper graph* shows all institutions, the *lower graph* focuses on one section of the tightly networked institutions in the *upper graph*. Although the MPI for Evolutionary Biology has published highly cited papers, this was not in cooperation with another institution in the network, so it is not included in the analysis. The map and network files are available at doi:10.6084/m9.figshare.1546480. Both files can be opened in VOSviewer

**Fig. 4:**
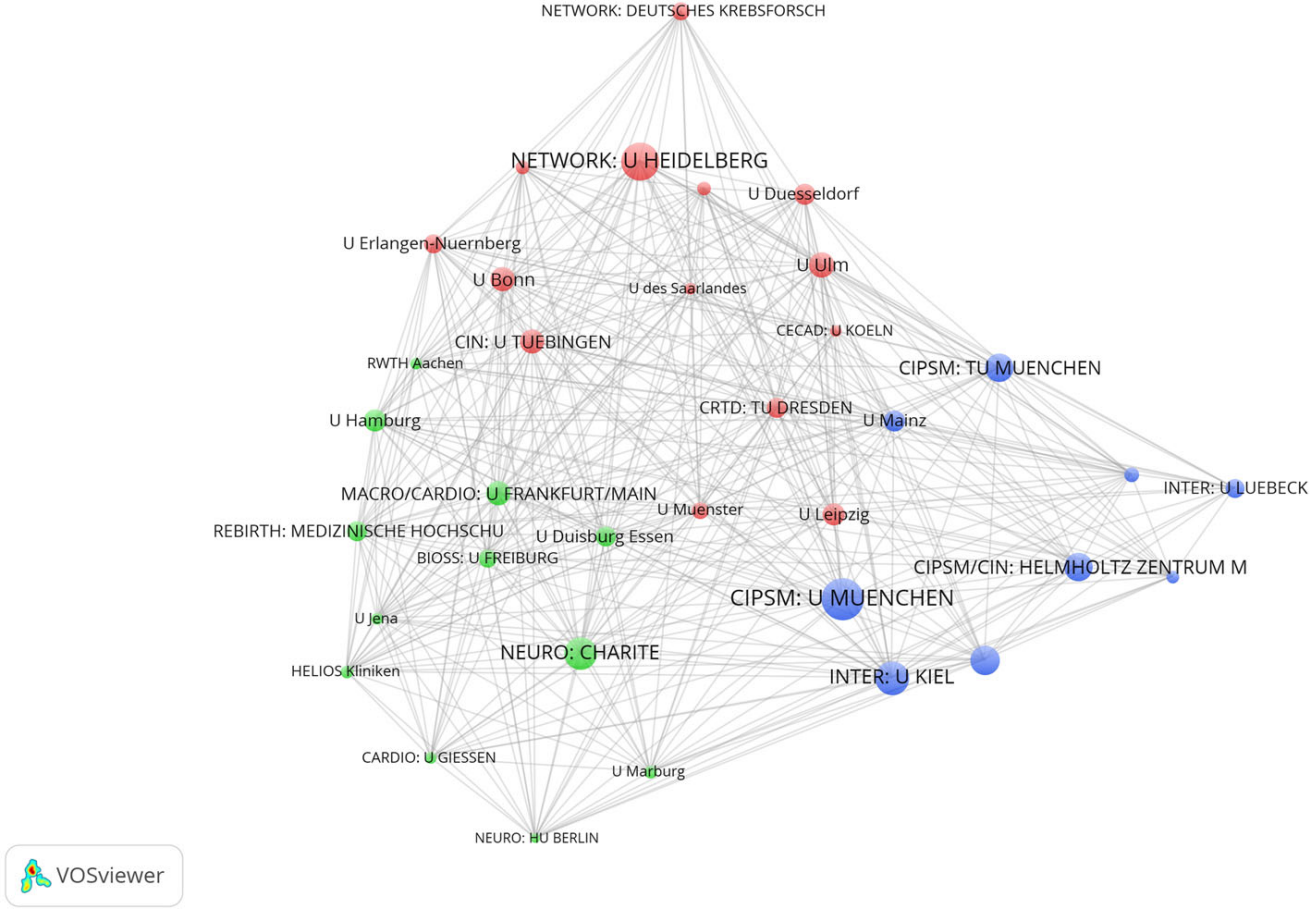
Sub-network of institutions from Fig. 3 which are tightly connected by co-authorships (life sciences, 2009–2011). Each institution has published a minimum of 25 papers with another institution

Figures 3 and 4 show the results for the years after the start of support under the Excellence Initiative. Figure 3 shows the network of the 308 institutions which have published at least one highly cited paper in cooperation with at least one other institution in Germany between 2009 and 2011. Figure 4 refers to the 35 institutions from Fig. 3 which have networked together particularly well (i.e. they belong to the group of network institutions which have published papers together with at least 25 institutions).

Figure 3 makes clear that the excellence institutions are well represented in the network. Among the ten institutions with the highest total link strength are eight excellence institutions: CIPSM: U München (456), NETWORK: U Heidelberg (396), NEURO: Charite (360), INTER: U Kiel (312), CIPSM: TU München (270), CIPSM/CIN: Helmholtz Zentrum München (258), MACRO/CARDIO: U Frankfurt am Main, and CIN: U Tübingen (236). Also in the sub-network of institutions from Fig. 3 which are tightly connected by co-authorships (see Fig. 4) there are a number of excellence institutions. However, neither of the two graphs makes clear that the excellence institutions within a Cluster of Excellence are also positioned with appropriate proximity to each other or are marked with the same cluster colour. Against this background, additional results are presented for the years 2009–2011 in Table 3.

As the results in the table show, of the 58 excellence institutions 43 are considered in the network and 15 are not. Compared with the years 2003–2005, the number of excellence institutions in the network has therefore increased by only one institution. The average institutions per network cluster ratio for the years 2009–2011 is 0.77. This means that the ratio has increased compared with the years 2003–2005: accordingly, the excellence institutions of a Cluster of Excellence between 2009 and 2011 are on average distributed across a larger number of network clusters than between 2003 and 2005. This result does not match the expectations of a greater concentration of excellence institutions in corresponding network clusters.

### Results for natural sciences

The Clusters of Excellence in the field of natural sciences and the associated institutions are shown in Table 1. In natural sciences with eight Clusters of Excellence, fewer clusters have been supported than in the life sciences with 12 clusters. It is also noticeable that significantly more institutions in natural sciences are represented in several Clusters of Excellence than those in life sciences. For example, U München is involved in three Clusters of Excellence (NIM, UNI, and MAP).

**Fig. 5:**
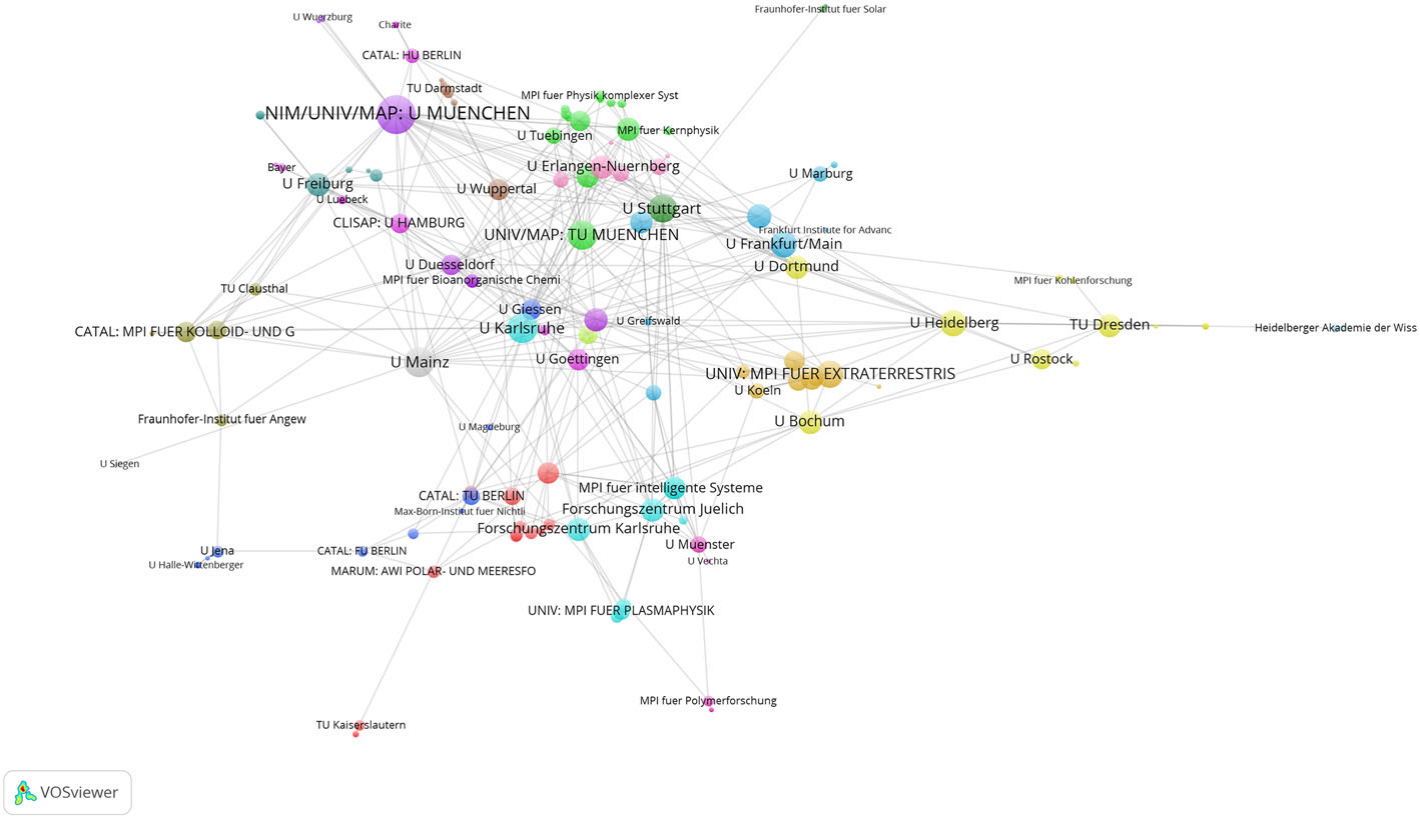
Network of institutions by co-authorships (natural sciences, 2003–2005). Although U Augsburg, the Helmholtz-Zentrum Geesthacht and the Fritz-Haber-Institut have published highly cited papers, this was not in cooperation with another institution in the network, so they are not included in the analysis. The map and network files are available at doi:10.6084/m9.figshare.1546480. Both files can be opened in VOSviewer

**Fig. 6:**
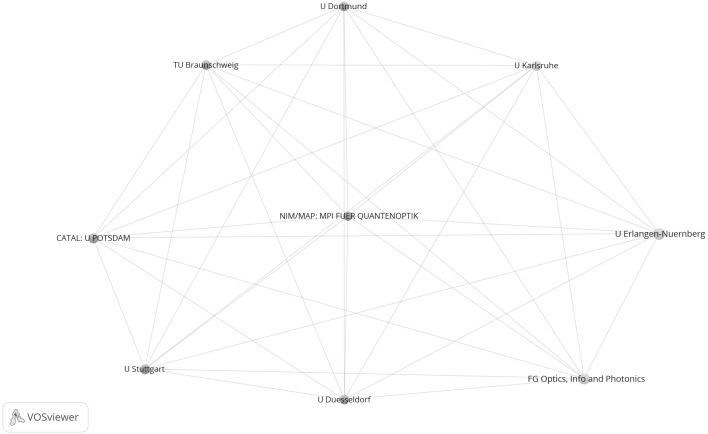
Sub-network of nine institutions from Fig. 5 which are tightly connected by co-authorships (natural sciences, 2003–2005). Each institution has a minimum of 8 papers in cooperation with another institution

Figure 5 shows the network of 132 institutions; it is based on the highly cited papers from the years 2003 to 2005. Because the whole network is already well recognizable in the Figure, no additional extract was generated from the Figure in VOSviewer (as in Fig. 1). With a total link strength of 52, U München is the best networked institution in Fig. 5. It is true that a number of excellence institutions are recognizable in the figure (e.g. U München and TU München); however, the arrangement of the nodes in the space and the colour of the nodes do not suggest a clustering of institutions as is determined by the Clusters of Excellence. Figure 6 shows the sub-network of tightly connected institutions from Fig. 5 (identified with the k-core technique). In this network, too, no structure can be detected corresponding to the Clusters of Excellence. Two of the institutions shown in Fig. 6 (MPI für Quantenoptik and U Potsdam) each belong to a different Cluster of Excellence (although they are positioned here in one network cluster).

As Fig. 5 does not reveal the entire structure of the network, some key figures on the network are given in Table 4 in addition. As the numbers in the table show, of the total of 43 excellence institutions 28 (65 %) appear in the network; 15 institutions were not included in the network analysis. Accordingly, in comparison with life sciences with 72 %, in the natural sciences network fewer excellence institutions are represented in the years 2003–2005. As Table 4 also shows, the institutions per network cluster ratio across all clusters is 0.69.

**Table 4:** The number of excellence institutions which appear or do not appear in the networks (Figs. 5, 6, 7, 8)

Cluster of excellence	Number of institutions	Number of institutions within network	Number of institutions not in network	Number of network clusters	Institutions per network cluster
2003–2005					
NIM^b^	7	3	4	2	0.67
MATHE	2	1	1	1	1.00
OCEAN	4	2	2	1	0.50
UNIV	9	7	2	5	0.71
MAP	5	4	1	2	0.50
CLISAP^b^	4	2	2	2	1.00
MARUM	6	4	2	4	1.00
CATAL^b^	6	5	1	3	0.60
Total	43	28	15		0.69^a^
2009–2011					
NIM	7	6	1	3	0.50
MATHE^c^	2	1	1	1	1.00
OCEAN	4	2	2	1	0.50
UNIV	9	9	0	5	0.56
MAP	5	5	0	2	0.40
CLISAP^c^	4	2	2	2	1.00
MARUM^c^	6	3	3	2	0.67
CATAL	6	6	0	2	0.33
Total	43	34	9		0.54^a^

Also, the table shows the number of network clusters in which the institutions of a Cluster of Excellence are represented and how the ratio of institutions per network cluster looks

^a^The average of the ratios of institutions per network cluster is calculated as a harmonic mean

^b^Although U Augsburg (NIM), the Helmholtz-Zentrum Geesthacht (CLISAP) and the Fritz-Haber-Institut (CATAL) have published highly cited papers, this was not in cooperation with another institution in the network, so they are not included in the analysis

^c^Although the MPI for Mathematics (MATHE), the Helmholtz-Zentrum Geesthacht (CLISAP), the MPI for Marine Microbiology (MARUM) and the Jacobs University Bremen (MARUM) have published highly cited papers, this was not in cooperation with another institution in the network, so they are not included in the analysis

**Fig. 7:**
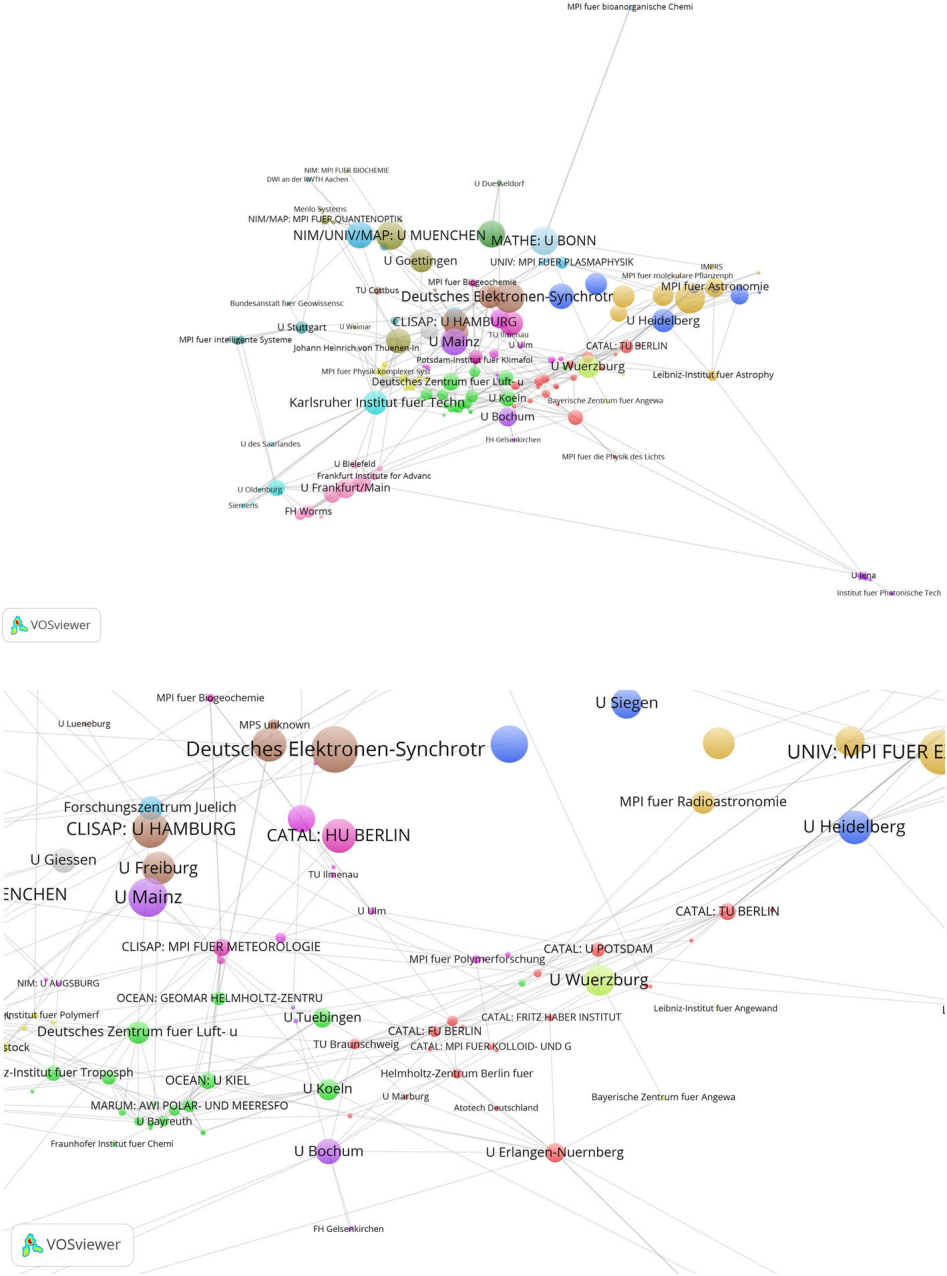
Network of institutions by co-authorships (natural sciences, 2009–2011). Whereas the *upper graph* shows all institutions, the *lower graph* focuses on one section of tightly networked institutions in the *upper graph* (network cluster 1 with five institutions in CATAL). The map and network files are available at doi:10.6084/m9.figshare.1546480. Both files can be opened in VOSviewer

Figure 7 shows the network of institutions in natural sciences for the years 2009–2011. Whereas the upper graph shows all institutions, the lower graph focuses on one section of tightly networked institutions in the upper graph. The network is based on 171 institutions which have published highly cited papers in cooperation with at least one other institution in Germany. Among the ten institutions with the highest total link strength are six excellence institutions (145: MPI for Extraterrestrial Physics, 128: U München, 128: U Bonn, 106: MPI for Physics, and 105: U Hamburg). As a closer inspection of the network reveals, five institutions of the total of six institutions of the CATAL Cluster of Excellence have been assigned to a single network cluster, so the lower graph of Fig. 7 focuses on the relevant extract in the network which relates to the excellence institutions in CATAL.

**Fig. 8:**
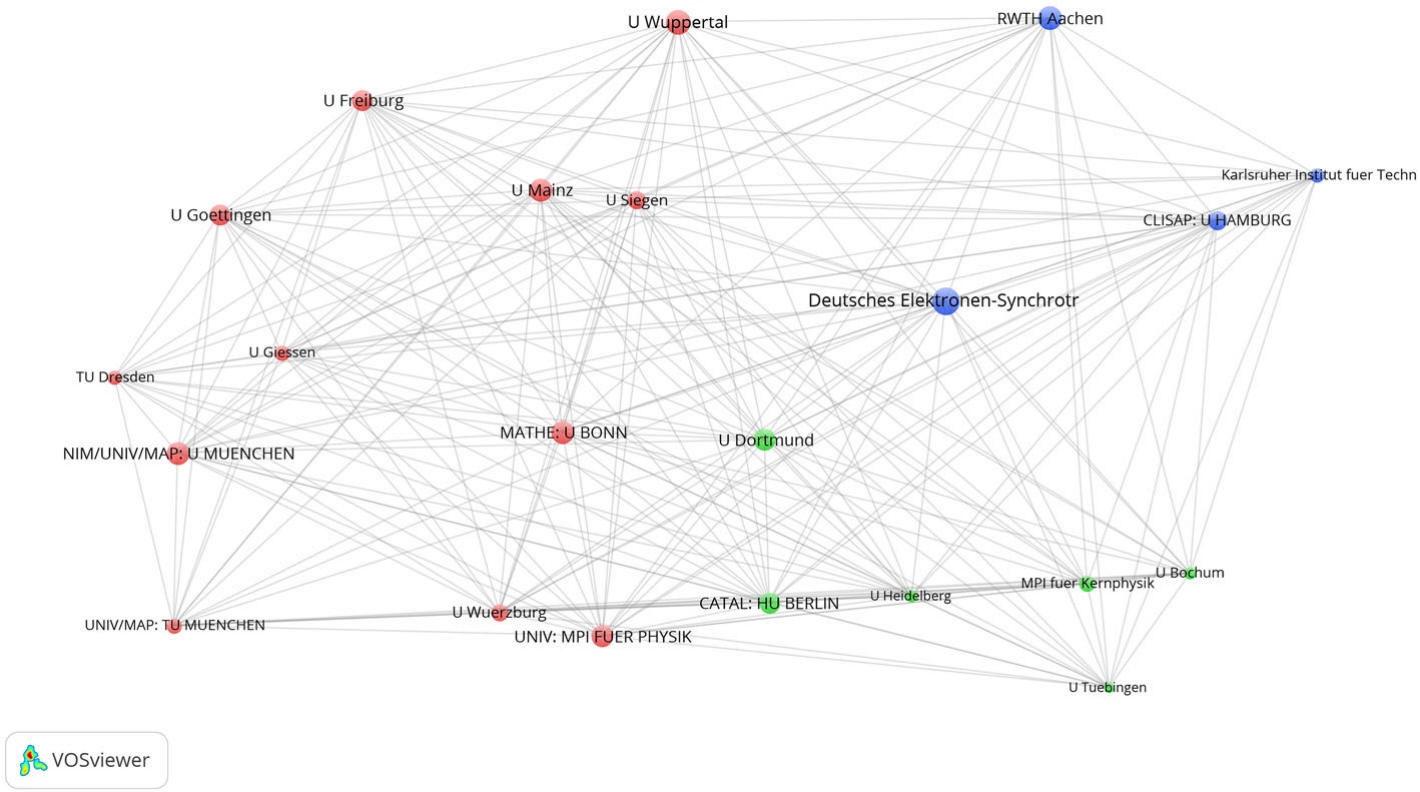
Sub-network of institutions from Fig. 7 which are tightly connected by co-authorships (natural sciences, 2009–2011). Each institution has a minimum of 16 papers in cooperation with another institution

Figure 8 shows the closely cooperating sub-network of the 22 institutions which have published together at least 16 times. Even if some excellence institutions can be recognised in this sub-network, the network does not reveal the structure determined by the Clusters of Excellence.

In addition to the two Figures, Table 4 gives some key figures on the entire network in Fig. 7. Of the total of 43 excellence institutions, 34 (79 %) are present in the network. This is significantly more institutions than in the period before establishment of the Clusters of Excellence (65 %). Accordingly, the institutions per network cluster ratio across all clusters is, with 0.54, also significantly lower than the ratio for the years 2003 to 2005, with 0.69.

## Discussion

As in a number of other countries as well, efforts have been made in Germany in recent years to strengthen research excellence through additional research grants and through competition between the institutions. Within the framework of the Excellence Initiative, significant additional resources, related to three funding lines, have been injected into the German science system. In the present study, the attempt has been made to measure the effect of one of these funding lines aimed at supporting Clusters of Excellence. As the particular purpose of this funding line is to promote research excellence and research cooperation, institutional networks have been analysed in terms of the papers among the 1 % most frequently cited papers in their subject category and year of publication. As the literature analysis of Bornmann (2014) shows, most studies which have identified highly cited papers across the *x* % most frequently cited papers are based on the 1 % of the most frequently cited papers.

Because the DFG has assigned the Clusters of Excellence in its database according to subject areas, the appropriate network analyses were undertaken in this study for life sciences and natural sciences and compared with the structures of the Clusters of Excellence. The highly cited papers from the MPG in-house database were assigned to these two subject areas by using subject-specific journal sets. In order to be able to identify the effect of establishing Clusters of Excellence on the bibliometric networks, networks were created for the period 2003–2005 (i.e. before the launch of the Excellence Initiative) and for the period 2009–2011 (i.e. after the launch of the Excellence Initiative in 2006 or 2007). It would have been anticipated that establishment of the Clusters of Excellence would be reflected in the data.

As the results of this study have shown, the outcomes yielded for life sciences and natural sciences differ from each other. Whereas life sciences display hardly any effect of the establishment of Clusters of Excellence on the networks, this was not true of natural sciences, where an effect was shown. After establishment of clusters in natural sciences, not only were more excellence institutions represented in the network than before establishment but these institutions were also spread across fewer network clusters. Also, almost the whole of one cluster of excellence (CATAL) was found in one bibliometric cluster. Thus, the bibliometric results in natural sciences reflect the structure given by the Excellence Initiative better than the bibliometric results in life sciences. However, one should not conclude from the results of the study that the life sciences in Germany are not successful in doing excellent research and to collaborate intensively. This study either undertook an evaluative bibliometric study which measured the general performance of life sciences in Germany, nor investigated the broad spectrum of their institutional collaborations. Life sciences in Germany may be successful independently of the establishment of the Clusters of Excellence and their collaboration activities could especially focus on international relations. An empirical study using bibliometric data to investigate a wide-ranging funding programme will always be subject to certain limitations. The comments below identify four key limitations of the study: (1) This study dealt with a very specific analysis of the support programme, which ought to be amplified by further evaluations. These further evaluations should not rely solely on bibliometric data, but should also include other research data (with which, for example, trends in technology or software developments in the Clusters of Excellence could be measured) and interviews (with involved scientists and other stakeholders).

(2) The effect of the Clusters of Excellence on the bibliometric networks was measured using publications from the years 2009 to 2011. As the clusters were established in 2006 or 2007, the period between establishment and measurement could be seen as too short. The research for papers first needs to be carried out and then published before it can be measured after inclusion in literature databases (e.g. in WoS). This is why this study should be repeated in a few years’ time in order to check whether the outcomes obtained here can be confirmed or not. In these studies it should also be checked whether the results are robust even if different indicators and statistics are used (than those which have been considered here). For example, as indicator of scientific excellence the 10 % most frequently cited papers could be used in addition to the 1 % most frequently cited. (3) The Clusters of Excellence also include institutions, which are primarily not really research institutions (e.g. the XLAB—Experimental Laboratory for Young People in Göttingen). These institutions were included in the Clusters of Excellence because the key feature of the Excellence Initiative is not just research excellence but also knowledge transfer into society (Bornmann 2013). These institutions are considered in this study firstly because it is hardly possible to determine conclusively whether an institution does not engage in any research at all. The second reason is that it ought to be possible to assume that a publication from a Cluster of Excellence would name all the institutions involved in the cluster.

(4) In this study, the highly cited papers published by the German institutions were assigned to two subject areas (life sciences and natural sciences) by using WoS journal sets. Although this is a standard approach in bibliometrics (Bornmann et al. 2014), it has the disadvantage that papers published in multi-disciplinary journals (e.g. *Science* and *Nature*) are not considered. These papers are assigned by Thomson Reuters to a multi-disciplinary category. Thus, it is possible that institutions which have been investigated in this study have jointly published more highly-cited publications in natural sciences or life sciences than considered here. Since larger datasets are not available from Thomson Reuters, where each single publication from these journals is reliably categorized to subject categories, this limitation of the study is scarcely avoidable.

## Conclusions

With the introduction of the Excellence Initiative in 2006, a total of €1.9 billion were made available for three funding lines between 2006 and 2011 (1: Graduate schools to promote early career researchers; 2: Clusters of Excellence to promote top-level research; 3: Institutional strategies to promote top-level university research). The second line which was the target of this study supports subject-specific cooperation between German institutions. Since the German chancellor and the minister-presidents of the states have reached an agreement concerning the succession of the Excellence Initiative after 2017, it is important that the success of the previous funding lines is empirically investigated. Future funding lines should be developed against the backdrop of these empirical results. Since this study is limited to a bibliometric analysis of one funding line only, it should be seen as one of the first empirical steps which should encourage further research (including all funding lines as well as further data, methods, and techniques). In general, it is amazing to observe (not only in Germany) that specific funding instruments have been developed, but the (long-term) effects of these instruments have been scarcely investigated. We will see whether the wide-ranging evaluation of the Excellence Initiative and its effects on the German science system which are expected to be published in 2015 or 2016 will bring about practically useful results. As the results of this study show the results can be ambivalent.
